# Establishment of an In Vitro System of the Human Intestinal Microbiota: Effect of Cultivation Conditions and Influence of Three Donor Stool Samples

**DOI:** 10.3390/microorganisms9051049

**Published:** 2021-05-13

**Authors:** Regina Haindl, Julia Engel, Ulrich Kulozik

**Affiliations:** Chair of Food and Bioprocess Engineering, ZIEL—Institute for Food & Health, Technical University of Munich, Weihenstephaner Berg 1, 85354 Freising, Germany; julia.engel@tum.de (J.E.); ulrich.kulozik@tum.de (U.K.)

**Keywords:** continuous flow fermentation, donor stool, short-chain fatty acids, richness, diversity, 16S rRNA sequencing

## Abstract

Fecal microbiota transplantation (FMT) is an alternative method for the treatment of gastrointestinal diseases with a high recovery rate. Disadvantages are ethical concerns, high donor requirements and the low storability of stool samples. The cultivation of an in vitro microbiota in a continuous bioreactor was established as an alternative to FMT to overcome these problems. In this study, the influence of the system parameters and donor stool characteristics was investigated. Each continuous colonic fermentation system was inoculated with feces from three different donors until a stable state was established. The influence of the fermentation conditions on the system’s behavior regarding cell count, metabolic activity, short-chain fatty acid profile and microbiota composition as well as richness and diversity was assessed. Cultivation conditions were found to affect the microbial system: the number of cells and the production of short-chain fatty acids increased. The abundance of Actinobacteria and Firmicutes decreased, Bacteroidetes increased, while Proteobacteria and Verrucomicrobia remained largely unaffected. Diversity in the in vitro system decreased, but richness was unaffected. The cultivation of stool from different donors revealed that the performance of the created in vitro system was similar and comparable, but unique characteristics of the composition of the original stool remained.

## 1. Introduction

The human gut hosts a complex, very diverse and large ecosystem called the intestinal microbiota. Due to the high retention time of food and, therefore, the high amount of nutrients, the colon has the highest abundance of microbial cells with up to 10^11^ CFU mL^−1^ and 400–1000 different species [1,2,3]. One function of the gut microbiota is the generation of a wide range of bioactive compounds that result from the bacterial transformation of otherwise indigestible food components. Bacterial metabolites, such as short-chain fatty acids (SCFAs), play a role in the defense against pathogens and regulate glucose homeostasis as well as lipid metabolism [4,5,6].

The microorganisms in the human intestinal microbiota belong to the phyla Firmicutes, Bacteroidetes, Actinobacteria, Proteobacteria and Verrucomicrobia. Firmicutes and Bacteroidetes represent the most abundant phyla, each constituting 40 to 50% of total bacteria. Actinobacteria (around 2.5%), Proteobacteria (0.1–1%) and Verrucomicrobia (~0.1%) are less abundant [1,7]. Variety, diversity and cell count have a major impact on the human health and immune system. Alterations in microbiota composition, e.g., a decrease in diversity and compositional changes, the so called disbalance, are related to several diseases [8]. These diseases are linked with the digestive system [9,10,11,12,13,14]. Furthermore, the microbiota has been reported to have an impact on non-digestive diseases, such as Parkinson’s disease [15] or cancer [16]. For example, a decrease in microbial diversity and cell count is linked to *Clostridium difficile* infections [12,17].

The standard therapy for *Clostridium difficile* infections (CDI) is treatment with antibiotics, such as vancomycin or metronidazole [18], but this can result in the occurrence of relapses and multi-resistant strains. Fecal microbiota transplantation (FMT) offers an alternative therapy. Here, stool from a healthy donor is transferred into the patient to restore the diversity and balance of the microbial community in the gut. The donor must be healthy, must not have taken antibiotics before FMT and must run through extensive screening in advance. For the transplantation, the stool is mixed with saline or medium, homogenized and filtrated, and the supernatant is transferred into the patient’s colon by means of a colonoscopy, nasogastric tube or enema. To increase the chance of recovery, the transfer of microorganisms as well as other molecules, e.g., short-chain fatty acids, is required [19,20]. Then, the success of FMT has been shown to result in recovery rates of up to 90% [21,22,23]. Nevertheless, the extensive screening, safety and ethical concerns, acceptability and the lack of a standardized treatment procedure are the main constraints of the method. To date, different approaches for the enhancement of the FMT treatment method include applications of frozen or freeze-dried stool samples [24,25,26]. Further, the cryopreservation of artificial microbiota was studied [24].

This study is part of a concept aiming at the generation of transferrable material in vitro to overcome concerns and challenges related to traditional FMT. The expectation is that by mimicking gut conditions in vitro, the production of controlled and stable artificial colonic microbiota would be possible. In vitro models have already been applied successfully to reveal the mechanistic effects of probiotics, drug absorption and transport [27,28,29,30,31,32,33]. Advantages of in vitro models are uncomplicated set-ups, operation and possibilities for variation and adaption of the system and, furthermore, the absence of ethical considerations. Several systems aiming to mimic cultivation conditions similar to the human colon have already been studied [24,34,35,36,37,38,39,40,41,42]. They commonly have a gas sparging system supplying CO_2_, N_2_ or forming gas to ensure anaerobic conditions. The temperature is normally set to +37 °C. Nutrients are supplied by a complex growth medium adapted in accordance with Macfarlane and Gibson [43]. To simulate the passage of food, continuous flow systems were applied with retention times of mainly 24 h [36,37,39], similar to the mean retention time in the human colon [44]. The current models worked with a vessel volume of 9 [42] to 400 mL [36]. For FMT, the volume of the used stool suspensions varied between 200 and 500 mL, with larger volumes yielding a better outcome [21,22]. To date, only models with a vessel volume too low to continuously harvest higher amounts of infusion material for FMT or complex multi-stage systems requiring high maintenance efforts and complicated set-ups have been tested.

In this study, we combined different parts of existing cultivation models to establish a less complex, easy to use system by choosing a single-stage cultivation bioreactor without cell retention with a higher vessel volume of 850 mL. This should enable the generation of larger amounts of infusion material for FMT by the continuous removal of cell suspension in a flow-through mode according to the feed volume flow at the system’s inlet to keep the volume constant over time. Nevertheless, it is unknown whether an amplification of the vessel volume and a simplification to a single-stage system without cell retention still generates a culturable and stable community with a similar richness and diversity. The approach was as follows: the impact of the cultivation conditions themselves as well as the individual characteristics of the donor stool were observed until the formation of a stable state in composition and number of cells was achieved. The open question was in which way the cultivation system itself, but also the individual donor stool characteristics, affects the microbiota characteristics in the continuously harvested cell suspension. The aim was to demonstrate the technical feasibility of the production of a culturable, stable community with a high diversity and richness of representative gut bacteria. Furthermore, the differences that may occur by the cultivation of three various stool samples were identified. The bioreactor system established in this study may be preferentially used for the cultivation of artificial colonic microbiota instead of direct FMT in later studies.

## 2. Materials and Methods

### 2.1. Donor Stool

For this study, stools from three different donors of one defecation each were used. The donors were chosen and tested regarding the criteria of Terveer et al. [45]. To minimize external influences, the donors were the same age group (donor A: 28 years old; donor B: 25 years old; donor C: 27 years old), ethnic group (Caucasian) and social environment. All donors had a normal body mass index (BMI) of 23 (donor A) and 21 (donors B and C) and ate a Western diet. Their last antibiotic treatment was at least 12 months ago. The stool was obtained in house and stored immediately at −80 °C prior to the experiments. To prevent a potential transfer of diseases, the donors’ stool was tested for bacterial, viral and eucaryotic pathogens that are relevant for in vitro cultivation by the Institute for Medical Microbiology and Hygiene, University of Regensburg.

### 2.2. Cultivation Medium

For the preparation of inoculum and the cultivation itself, a medium mimicking the chyme in the human colon was used. It was adapted according to Macfarlane and Gibson [43] and called Continuous Flow Fermentation Medium (CFF) in the following. As shown in Table 1, the medium was prepared from six different stock solutions, where stock solution 1 was mixed with 800 mL double distilled H_2_O. Stock solution 2 and 3 were each mixed with 50 mL, and stock solution 4 was diluted in 100 mL. Solution 2 was subsequently filtrated with a 0.22 µm filter, whereas solution 1 and 4 were each autoclaved. For the preparation of solution 5, hemin (0.05 g) was dissolved in 1 mL 1 M NaOH and filled up to 100 mL with ultrapure H_2_O. Solution 6 was produced by mixing 0.1 g Vitamin K_1_ with 20 mL 95% ethanol. Solution 5 and 6 were stored in the refrigerator until use. Solution 3 was mixed, stirred and centrifuged using an Allegra X15R centrifuge (Beckmann Coulter Inc., Brea, CA, USA; 20 min, 6000× *g*, +4 °C). The supernatant was subsequently filtrated (0.22 µm) and joined with solutions 1, 2, 4, 5 and 6 under sterile, anaerobic conditions. The final medium was stored at +4 °C and used within 72 h.

### 2.3. Fecal Inoculum

For all experiments, the stool from one defecation of each donor was used. The preparation of inoculum was adapted in accordance with McDonald [36] and is similar to the preparation of infusion suspensions for FMT [21]. Therefore, a 10% (*w*/*v*) fecal slurry was prepared by mixing 30 g of stool with 300 mL CFF medium and homogenizing it for 15 s (Melissa, Adexi, Skødstrup, Denmark). To remove large food residues, the mixture was centrifuged (Allegra X15R, Beckmann Coulter Inc., Brea, CA, USA; 180× *g*, +4 °C, 10 min), and 120 mL of the resulting supernatant was used as the inoculum for each experiment of this study.

### 2.4. In Vitro Cultivation System 

The set-up of the cultivation system was adapted in accordance with already existing systems with the focus on an easy set-up and handling as well as a sufficient generation of transferable material [36,37,39]. Therefore, a continuous system without cell retention was chosen to constantly generate an output of a transferable slurry without a high need for constant surveillance and service. Further, a vessel volume of 850 mL was chosen. All cultivation experiments were performed in a *BioStat B* bioreactor (Sartorius AG, Göttingen, Germany). Cultivation was started by transferring 120 mL of inoculum sterile into the glass vessel containing 730 mL of anaerobic CFF medium. Anaerobic conditions in the bioreactor were maintained by sparging continuously with 8 ccm forming gas. The process conditions were adapted to the human colon by setting the temperature to +37 °C. Furthermore, the pH was adjusted to 6.5 [46] and regulated with 1.25 M NaOH and 0.5 M HCl, and the broth was stirred at 200 rpm. The turbidity was measured by an internal sensor to assess the cell density. Following a batch start-up phase of 24 h to allow the low number of cells in the inoculum to grow, the system was set to a continuous mode after 24 h of processing time by adding fresh CFF medium (automatic inflow of 0.5 mL min^−1^) and harvesting cell broth in the same amount. As a result, the mean retention time was 28.3 h, which is comparable to the human colonic transit time [44]. All cultivations were run for 120 h without interruption. At various time points during cultivation, cell broth was pumped anaerobically into prepared sample tubes (Greiner Bio-One, Kremsmünster, Austria). The cell broth was either used immediately (analysis of cell count) or stored at −80 °C for further analysis (analysis of SCFAs and sequencing). Storage under these conditions did not affect the analytical results.

### 2.5. Analysis of Facultative Aerobic and Anaerobic Cell Count

The numbers of anaerobic and facultative aerobic colony forming units (CFUs) were analyzed separately. Therefore, samples were collected and diluted with 0.25 strength Ringer’s solution and either plated on Wilkins–Chalgren Anaerobe agar plates (anaerobic cell count) or Plate Count agar plates (facultative aerobic cell count). For each point of time, up to three dilution factors and at least four plates per dilution were plated. The plates were incubated either aerobically or anaerobically for 48 h at +37 °C, and only plates with 30 to 300 colonies were included in the analysis. The number of CFUs N per mL of sample was calculated according to the equation (Equation (1)). Here, *c* is the sum of colonies of the subsequent dilutions with *n*_1_ the number of colonies in the less diluted solution, and *n*_2_ is the number of colonies in the more diluted solution.

(1) $$N=\frac{c}{n_{1}+\left(0.1\cdot n_{2}\right)}$$

### 2.6. Analysis of Short-Chain Fatty Acids by Means of High-Performance Liquid Chromatography

The concentration of sugars (glucose and galactose), metabolic intermediates (succinate and lactate), as well as the main short-chain fatty acids (SCFAs) acetate, propionate, butyrate and isovalerate, were identified and measured by means of high-performance liquid chromatography (HPLC). The HPLC unit (Agilent Technologies Inc., Santa Clara, CA, USA) was equipped with an *Aminex HPXH-87H* ion exclusion column (Bio-Rad Laboratories, Hercules, CA, USA) and a refractive index detector *G1362A* (Agilent Technologies Inc., Santa Clara, CA, USA). Separation was performed with 0.0005 mol L^−1^ H_2_SO_4_, 0.45 mL min^−1^ flow rate and 20 to 100 µL injection volume. Before injection, the samples were centrifuged (Hermel-Z233 M-2, Hermle Labortechnik GmbH, Wehingen, Germany; 6000× *g*, +20 °C, 30 min), and the supernatant was used after an additional filtration (0.22 µm). The metabolites were identified and quantified using external standards (Sigma Aldrich, Saint Louis, MO, USA) and the *Agilent ChemStation Instrument 1 offline* software (Agilent Technologies Inc., Santa Clara, CA, USA).

### 2.7. Microbial Profiling by Means of 16S rRNA Sequencing

The composition of the microbial community, richness and diversity were characterized in the stool samples and at several points of time during cultivation by sequencing the V3/V4 region of 16S rRNA. High-throughput 16S rRNA gene amplicon sequencing was performed by an external lab (Microbiome Core Facility, ZIEL-Institute for Food and Health, TU Munich) according to the protocol described by Reitmeier et al. [47]. After receiving the raw data, they were preprocessed using the *IMNGS* pipeline [48]. Before analysis, operational taxonomic units (OTUs) with a relative abundance <0.25% across all samples were removed to prevent the detection of spurious OTUs [49]. Furthermore, five nucleotides were trimmed on the 5′ and 3′ ends for the R1/R2 read, and the expected error rate was set to 3 (trim score 3). Only nucleotides with a read length between 300 and 600 were considered. Analysis of alpha diversity and taxonomy was performed by the provided R script *Rhea* [50,51]. The evaluation of alpha diversity richness, representing the total number of OTUs in the community and the Shannon effective index, which accounts for the evenness and abundance of species in the community, was analyzed automatically by the software.

### 2.8. Statistical Analysis

In this study, all analyses were repeated at least in triplicate. The arithmetic mean $\bar{x}$ represents the mean value of the number n of all samples *x_i_*. The distribution of the values was calculated from the standard deviation *s* due to the random error. All data, graphs and tables in the following show arithmetic means ± standard deviations. Statistical significance was tested using a one-way ANOVA (*p* ≤ 0.05) followed by a Tukey post hoc analysis conducted with the OriginPro 2019 software (OriginLab Corporation, Northampton, MA, USA).

## 3. Results and Discussion

In this study, an in vitro system representing the human intestinal system was developed to cultivate the microbiota of three different donors until a stable state was formed. Therefore, each continuous colonic fermentation system was inoculated with feces from one of the three different donors that originated from one defecation each. After 24 h of processing time, the fermentation was switched to continuous mode by adding fresh medium on the one side and removing broth in the same amount. The whole system was run until a stable state according to the criteria described below was established. The influence of the cultivation parameters on the system’s behavior was investigated regarding cell count, metabolic activity and SCFA production. Additionally, microbiota composition, as well as the richness and diversity of the microbial community, was assessed.

Further, the cultivation of all three donor samples was compared to reveal whether the obtained system is influenced by the process parameters, the donor characteristics or both.

### 3.1. Characterization of Donor Stools

Three donors were selected for the study. They shared a common social and ethnical background as well as a comparable diet. Each donor stool originated from one defecation. The stool was tested for cell count, metabolic profile and microbial diversity. There were no significant differences (*p* ≤ 0.05) in anaerobic or aerobic cell count among the three donors (Table 2). 

**Figure 1:**
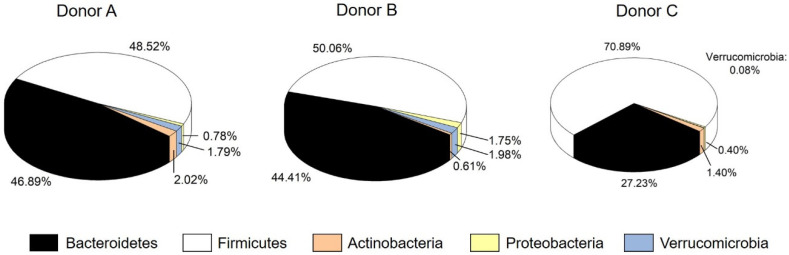
Relative cumulative abundance of the five major phyla in each stool sample.

The cell count of facultative aerobes was between 0.7 ± 0.4 × 10^5^ (donor A) and 10 ± 9 × 10^5^ CFU mL^−1^ (donor B). The high variation, especially in the stool of donor B, may be due the individual availability of aerobic growth of the microorganisms. The anaerobic cell count was more homogenous and between 2 ± 0.02 × 10^8^ (donor B) and 4 ± 3 × 10^8^ CFU mL^−1^ (donor A). Furthermore, the concentration of SCFAs in the stool was from 5.12 ± 0.14 (donor B) to 6.80 ± 0.47 mg mL^−1^ (donor A). The ratio for acetate: propionate: butyrate was comparable as well, with 3:1:1 for donor A, 3:2:1 for donor B and 3:1:2 for donor C. The concentration of propionate (2.00 ± 0.16 mg mL^−1^) in the stool of donor A was higher than for donors B and C. Higher values for propionate may indicate an overweight person, nonetheless, the BMI of donor A was normal [52]. Nevertheless, the overall metabolic profile represents three healthy donors with a normal contribution of SCFAs. Richness, Shannon effective index as well as the relative abundance of phyla were determined for the microbial profile. No difference among the donors was observed for richness between 100 (donor A) and 123 (donor B). Regarding diversity, represented by the Shannon effective index, a significantly higher value of 46.34 was identified for donor C. This coincides with an increase in Firmicutes and a decrease in Bacteroidetes and Verrucomicrobia for donor C. As Figure 1 shows, donor C hosted 70.88% Firmicutes and 27.22% Bacteroidetes. The abundance of Firmicutes for donor A (48.52%) and donor B (50.06%) was lower, while the abundance of Bacteroidetes was higher (44.41% for donor B; 46.89% for donor A). For Proteobacteria, donor B had a higher relative abundance of 1.75%. The abundance of Actinobacteria varied among all donors from 0.61 (donor B) and 1.40 (donor C) to 2.02% (donor A). The phylum of Verrucomicrobia was only present in low abundances between 0.08% (donor C) and 1.98% (donor B).

The different phyla were represented by several genera. Within the phylum of Actinobacteria, only *Bifidobacteria* were abundant. *Akkermansia* represented Verrucomicrobia, whereas *Escherichia* and *Shigella* can be found within the phylum of Proteobacteria. Bacteroidetes were represented by *Bacteroides*, *Parabacteroides*, *Prevotella* and *Alistipes*. The phylum Firmicutes were comprised of several genera of *Clostridium* as well as other genera, such as *Blautia*, *Dorea*, *Dialister*, *Faecalibacterium*, *Roseburia* or *Ruminococcus*. Not all detected genera were abundant in all stool samples. Among the genera with an abundance >1%, differences were detected regarding *Dialister*, *Eisenbergiella*, *Prevotella* and *Oscillibacter*. *Eisenbergiella* and *Oscillibacter* were not abundant in donor A, but in donors B and C. Donor B instead lacked *Dialister*. Donor C showed a significantly higher abundance in *Prevotella* than donors A and B. How these differences affect the formation of a stable system and the fermentation outcome is described below. Since all donors represent a healthy intestinal microbiome that is typical for a Western diet [7,53] and effects of cultural background, body mass or age were excluded, the main influencing factors on the established in vitro cultured microbiomes may be the system parameters themselves as well as the individual compositions.

### 3.2. Establishment of an In Vitro Microbiota

In the following, the establishment of the in vitro system is described by the cultivation of stool A. The same experiments were also performed for donors B and C but are not described in detail for the sake of clarity, since their behavior was comparable.

#### 3.2.1. Cell Count

**Figure 2:**
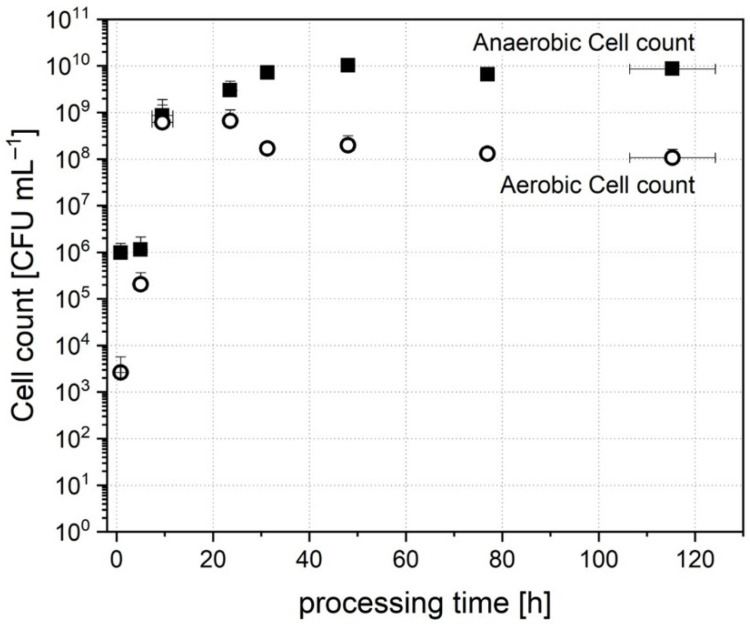
Cell count of anaerobic and aerobic cell count over processing time during cultivation of donor stool A.

The inoculum cell count level was 3 ± 0.7 × 10^6^ CFU mL^−1^ anaerobes and 3 ± 3 × 10^4^ CFU mL^−1^ facultative aerobes. After addition to the bioreactor and dilution with the medium, the cultivation cell broth contained a starting cell count level of 1 ± 0.6 × 10^6^ CFU mL^−1^ anaerobes and 3 ± 3 × 10^3^ CFU mL^−1^ facultative aerobes. Figure 2 shows the evolution of cell count over a processing time of 120 h.

The number of aerobic cells started to increase immediately after inoculation, and a peak was reached after 23.6 ± 1.3 h with a number of 6 ± 5 × 10^8^ CFU mL^−1^. The cell count then dropped slightly and stabilized subsequently. After 115.3 ± 8.9 h of cultivation, a stable number of 1 ± 0.5 × 10^8^ CFU mL^−1^ was reached.

In comparison, the number of anaerobic CFUs had a lag time of 5.0 ± 1.3 h in the beginning. After 9.5 ± 2.2 h, the cell count increased rapidly and reached 3 ± 2 × 10^9^ CFU mL^−1^ after 23.6 ± 1.3 h of processing time. At that point of time, it seemed that anaerobic cells had adapted to the conditions and then established a stable amount of 8 ± 2 × 10^9^ CFU mL^−1^ after 115.3 ± 8.9 h of processing time. The evolution of cell count and beginning of growth is in accordance with the detected turbidity (data not shown). The internal optical sensor detected a measurable total microbial growth after 5 ± 1.7 h of inoculation. Although the number of anaerobes in the inoculum was higher than that of facultative aerobes, the ratio of both was balanced after the first 9.5 ± 2.2 h. This dominance of facultative aerobes in the beginning may be explained by the fact that traces of oxygen were transferred into the reactor by the inoculation. Compared to the inoculation of gut at birth, this behavior seems similar. In vivo, the microbiota is also dominated by facultative aerobes, such as *Bifidobacteria* and *Lactobacilli*, after birth [54]. Regarding only cell count, the establishment in vitro is similar to the establishment of cells in the gut in early life. When growing up, the cell count increases further to an average human microbiota content of about 10^8^ to 10^11^ anaerobic CFU mL^−1^ [3]. Consequently, a comparable cell count was reached in this study and could be maintained during the processing time.

#### 3.2.2. Metabolic Profile and SCFA Production

**Figure 3:**
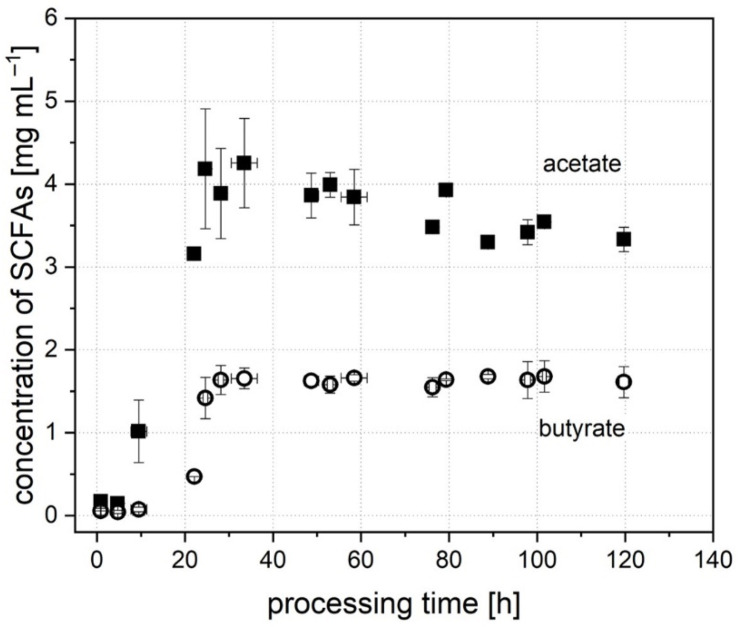
Concentration of the SCFAs, acetate and butyrate, throughout the processing time during the cultivation of donor A.

In addition to cell count, the concentration of metabolic intermediates and products was determined throughout the processing time to provide further data on the molecular composition affecting microbial growth and the behavior of individual groups. When starting the cultivation, only trace amounts of SCFAs and intermediates were found in the cell broth originating from the inoculum prepared from stool. In accordance with cell growth, metabolic activity started to grow after 8 to 22 h. The development of concentration of the major SCFAs, acetate, butyrate, propionate and isovalerate, is described in the following. As Figure 3 shows, a low concentration of 0.17 ± 0.08 mg mL^−1^ of acetate was contained in the broth after inoculation. Growth was detected after 10 ± 1.8 h of processing time, when the SCFA concentration had increased to 1.02 ± 0.38 mg mL^−1^. The highest concentration of acetate in the broth was detected after a processing time of 24 to 36 h. Here, a concentration of 4.14 ± 0.59 mg mL^−1^ at the peak of acetate production occurred. The amount then dropped slowly and stabilized after 76 ± 0.8 h to a stable concentration of 3.50 ± 0.22 mg mL^−1^.

In comparison, the concentration of butyrate (Figure 3) after inoculation was lower (0.06 ± 0.05 mg mL^−1^), and growth was detectable later, after 22 h (0.47 ± 0.00 mg mL^−1^). In contrast, the maximum was reached earlier compared to the concentration of acetate. Here, a concentration of 1.64 ± 0.18 mg mL^−1^ was established after 28 ± 0.6 h of processing time. The concentration of propionate at the start of cultivation was 0.10 ± 0.02 mg mL^−1^. It increased continuously until a stable concentration of 2.94 ± 0.13 mg mL^−1^ was reached after 78.0 ± 1.7 h. For isovalerate, the concentration after inoculation was not detectable (<0.01 mg mL^−1^), but increased after 24.3 ± 0.3 h and reached a stable concentration of 0.25 ± 0.04 mg mL^−1^ after 77.3 ± 1.7 h. To evaluate the in vitro system, the concentration of SCFAs was compared to studies from the literature. Schwiertz et al. [52] detected the concentration of SCFAs for healthy, overweight and adipose persons. Compared to these data, acetate, butyrate and isovalerate are in the range of a healthy person with a normal weight. Only the concentration of propionate was typical for an obese person. Bircher et al. [24] cultivated human microbiota in a 24 h batch system. They obtained similar concentrations for acetate and butyrate. The concentration for propionate was lower compared to the value of system A in this study. The values for systems B and C are in accordance with the findings of Bircher et al. [24]. This proves that the characteristics of the donor stool, e.g., a high propionate production by donor A, can also be found in the in vitro microbiome. Overall, the in vitro system represents a functional microbiome with a regularly working metabolism.

In the human intestinal microbiota, the ratio of acetate, propionate and butyrate is a marker for human health. Rowland et al. [55] and Macfarlane et al. [56] claim that a ratio from 3:1:1 (acetate: propionate: butyrate) to 10:2:1 as typically healthy. The in vitro microbiome developed in this study shows a ratio of 4:3:2. For the success of the in vitro alternative to FMT, not only the effective transfer of microorganisms is required. The transferred SCFAs also play a major role in the recovery of the patient [19]. Consequently, the in vitro system obtained here represents a healthy human microbiota regarding the SCFA ratio that may be further used for FMT.

In addition to the main SCFAs, the metabolic intermediates succinate and lactate were analyzed. These substances are metabolized to acetate or butyrate, e.g., by *Bifidobacteria* [57]. The production of succinate started after 23.6 ± 1.2 h up to a maximum of 0.98 ± 0.02 mg mL^−1^. The concentration was subsequently lowered to below 0.04 mg mL^−1^ after 77.4 ± 2.7 h. The progress was similar for lactate. First, a constant concentration of 0.12 ± 0.05 mg mL^−1^ was in the broth for the first 24 h until it dropped below a concentration of 0.02 mg mL^−1^. Facultative aerobic microorganisms, such as *Lactobacilli* and *Bifidobacteria*, are the main producers of succinate and lactate in the microbiome. Consequently, the production of metabolic intermediates is in accordance with the development of aerobic CFUs. The main growth occurs in the first 80 h. Afterwards, a stable system with a constant metabolic activity and lower levels intermediates is established.

#### 3.2.3. Definition of the Stable System

To compare and evaluate the in vitro system, a control value had to be defined. During cultivation, the value for SCFAs changed in the beginning but then plateaued, indicating stable state conditions. In this study, the stability of fermentation performance was defined as the point in time when the values for acetate, butyrate as well as propionate did not vary by more than 1% per h of processing time. For donor A, the stable system was reached after 29.2 ± 1.9 (acetate), 76.2 ± 1.0 (propionate) and 31.5 ± 0.3 h (butyrate). Overall, a stable state was reached after a maximum of 77 h. To verify this, a cultivation was run for 180 h of processing time. Here, as well, no shift in the factors described above was detected after 77 h. For further comparison and evaluation, averaged values were used in the stable system of 77 to 120 h.

#### 3.2.4. Microbial Composition and Diversity

The development of microbial composition, richness and diversity was determined by sequencing 16S rRNA gene amplicons. Stool A was from a healthy donor with a normal microbial distribution and diversity. In this stool, the major phyla were represented by Bacteroidetes and Firmicutes with a relative abundance of 46.89% and 48.52%, respectively. Actinobacteria (2.02%), Proteobacteria (0.78%) and Verrucomicrobia (1.79%) were present in lower relative abundance numbers. This human microbiome also hosted a richness of 100 and a diversity with a Shannon effective index of 21.42 (Table 2). When preparing the fecal inoculum, richness (103.40 ± 5.68) and diversity (23.35 ± 2.76) stayed constant. In contrast, the distribution of the phyla changed. As Table 3 shows, the relative abundance of Actinobacteria in the inoculum increased, while Proteobacteria and Verrucomicrobia decreased. The relative abundance of Bacteroidetes dropped to 1.59 ± 1.09%, while Firmicutes increased to 94.82 ± 0.75%.

This is probably due to the oxygen and shear stress sensitivity of the different phyla. During the preparation of the inoculum, the exposure to oxygen was kept as low as possible but could not be fully avoided. Further to that, by mixing and centrifugation, shear stress occurred that may have had an impact on the abundance of various groups of cells. Consequently, the abundance of Bacteroidetes as anaerobic microorganisms decreased, but could be fully restored during cultivation. Although there was a drop after inoculation, the abundance increased after 24.6 ± 0.6 h to 37.91 ± 1.65% and finally established with a value of 75.99 ± 1.78% in the stable system. To identify the ongoing microbial changes at the genera level, we defined clusters of sequences representing single microbial entities, known as operational taxonomic units (OTUs) that share at least 97% of genetic similarity based on the 16S rRNA V3/V4 region. In the stable system, the most represented genera with >1% relative abundance within the Bacteroidetes phylum are *Alistipes* and *Bacteroides*. They occur with a relative abundance of 6.41 ± 4.97% (*Alistipes*) and 68.17 ± 5.05% (*Bacteroides*).

In comparison, the relative abundance of the phylum Firmicutes in the inoculum is way higher (94.82 ± 0.75%). After inoculation, the abundance started to decrease to a value of 49.20 ± 2.12% after 24.6 ± 0.6 h. The final relative abundance in the stable system was 16.92 ± 1.96%. In the inoculum, the main representing genera within the Firmicutes phylum were *Blautia*, *Clostridium XIVa*, *Dialister*, *Faecalibacterium*, *Gemmiger*, *Lachnospiracea*, *Roseburia* and *Ruminococcus*. During cultivation, the abundance of *Blautia*, *Gemmiger*, *Lachnospiracea*, *Roseburia* and *Ruminococcus* lowered <1%. *Clostridium XIVa*, *Dialister* and *Faecalibacterium* were able to establish in higher abundances in the in vitro system. The abundance of *Clostridium XIVa*, represented with 1.95 ± 0.74% in the inoculum, was stable during cultivation (2.91 ± 0.42% in the stable system). The genera *Dialister* decreased in its abundance from 3.63 ± 0.15% in the inoculum to 1.06 ± 0.38% in the stable system. *Faecalibacterium*, which was reported to have a major influence on human health, was preserved [58]. This genus was abundant in the inoculum (7.53 ± 1.07%) as well as in the stable system (3.91 ± 0.67%).

The phylum Actinobacteria decreased after inoculation and was established with an abundance of 0.09 ± 0.05% after 77 h. This phylum is represented by the genus *Bifidobacterium*, which was present with an abundance of 2.24 ± 0.55% in the inoculum but decreased to a value of 0.05 ± 0.02% in the stable system. The phylum Verrucomicrobia showed a contrary behavior. While only a low abundance of <0.05% was observed in the inoculum, it increased after 48.0 ± 1.3 h to 1.10 ± 0.76% and finally stabilized at a value of 2.22 ± 0.72%. This phylum is represented mainly by the genus *Akkermansia*. The phylum Proteobacteria showed a varying abundance during cultivation. After inoculation, the abundance increased from 0.12 ± 0.14 to 11.37 ± 7.69% after 8.3 ± 2.6 h. Then, a decrease and afterwards stabilization in the stable system at an abundance of 4.38 ± 2.42% was observed. This peak in abundance can be explained since Proteobacteria are mainly facultative aerobe microorganisms. The trend in abundance is similar to the development of aerobic CFUs. Further, the production of acetate followed a comparable development, from which it can be concluded that most of the Proteobacteria in the system produced acetate. Here, the main producers were the genera *Escherichia* and *Shigella*, which were represented in the stable system at 2.34 ± 0.59%. The progress of the different phyla shown in this study is comparable to the formation of the microbiota after birth [59]. Here, the system was also dominated by facultative and aerotolerant bacteria, as Proteobacteria, Firmicutes and *Bifidobacterium*, in the beginning. Consequently, the formation of acetate was highest during the first 48 h. Afterwards, strict anaerobic, butyrate producing bacteria as representatives from the phylum Bacteroidetes started to grow and establish in the gut as well as in the in vitro system. Overall, in the in vitro cultivation system, the relative abundance of the phyla differed from the abundance in the donor stool. The value of Actinobacteria and Firmicutes was lower since Bacteroidetes, Proteobacteria and Verrucomicrobia have a higher abundance in vitro than in vivo in contrast. The decrease in Firmicutes may have been caused by the higher redox potential exceeding normal physiological levels in the in vitro system, which was also observed by Zihler et al. [60]. The change from an in vivo to an in vitro environment may possibly inactivate sensitive populations of bacteria or may also convert stressed microorganisms into an abundant, but unculturable state and will, therefore, be washed out from the system. Nevertheless, the aim was not to produce an exact copy of the in vivo system, but rather a culturable, stable community with a high diversity and richness of representative gut bacteria. After all, this aim was reached by generating a stable system comparable to the donor stool. On the other hand, the human intestinal microbiome is also not totally stable. In vivo variations even on a daily timescale level were observed [61]. Considering this, certain changes in the in vitro system, probably due to the cultivation parameters, would be acceptable. Our results are in accordance with the findings of McDonald [36], who also detected a comparable distribution of phyla when cultivating microbiota at pH 7.0. Similar observations were also made for a polyfermentor intestinal model [60]. In these works, both obtained an in vitro system with a reduced abundance of Firmicutes and an increase in Bacteroidetes as well as Proteobacteria. In addition to the microbial composition of the in vitro microbiota, richness and diversity were observed. For richness, a decrease to 11.00 ± 4.36 after 8.3 ± 2.6 h was distinguished. Afterwards, an increase after 24.6 ± 0.6 h to 96.33 ± 12.66 was noticed. In the stable system, a richness of 105.17 ± 5.71, comparable to the donor stool, was established. Diversity, characterized by the Shannon effective index, showed a development similar to richness. A decrease after 8.3 ± 2.6 h to 8.32 ± 4.22 was observed as well as an increase and further stabilization to a value of 17.28 ± 1.14. In comparison to the inoculum as well as the original stool, richness and Shannon effective index showed similar values. Consequently, richness as well as diversity can be restored with the cultivation method and parameters in this study.

### 3.3. Influence of Donor Sample on In Vitro Microbiota in the Stable System

The influence of the donor sample was tested by comparing all values in the stable system. For all samples, the stable system was defined as the change over cultivation time in concentrations of acetate, butyrate as well as propionate <1% per h of processing time. For donor A, the stable system was reached no later than 77 h. For donor B, the stable state was reached after 76 h, and for donor C after 74 h. Consequently, the averaged values of each factor of 77–120 h processing time were considered for a comparison.

Table 4 shows the value of all investigated factors in the stable system.

For cell counts, no significant differences were observed. All donor samples resulted in between 0.9 ± 0.2 × 10^10^ (donor A) and 1 ± 0.3 × 10^10^ CFU mL^−1^ (donor B) of anaerobic cells. For the concentration of SCFAs, an increase in the total amount from 8.29 ± 0.55 mg mL^−1^ for donor A to 9.22 ± 0.27 mg mL^−1^ for donor C was detected. In detail, no significant difference was observed for propionate. In contrast, the concentration of acetate, butyrate and isovalerate varied. For acetate, donor B showed a significantly higher value of 3.82 ± 0.14 mg mL^−1^. For butyrate, the concentration of 2.39 ± 0.15 mg mL^−1^ increased for donor C. In the in vitro microbiota of donor A, the concentration of isovalerate decreased significantly to 0.24 ± 0.04 mg mL^−1^. Under these conditions, the ratio of acetate:propionate:butyrate was 3:3:2 for donor A and 4:3:2 for donors B and C.

**Figure 4:**
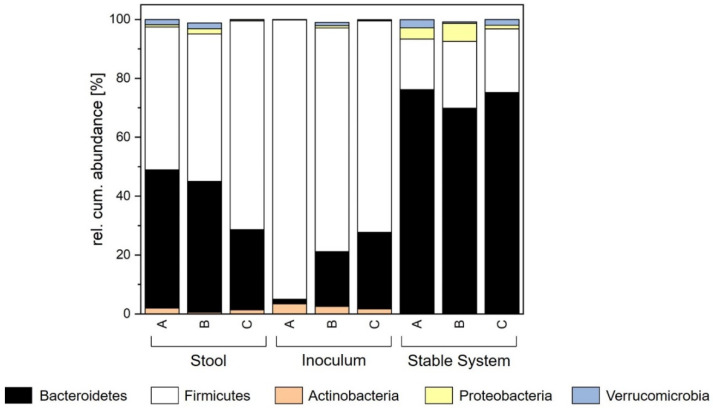
Comparison of relative cumulative abundance of the major phyla for stool, inoculum and stable system for each donor.

Diversity, represented by the Shannon effective index, was significantly higher in the system of donor B with a value of 24.40 ± 1.75. In terms of richness, all donors differed. Richness increased from 96.00 ± 7.07 (donor B) to 105.17 ± 5.71 (donor A) and 115.00 ± 2.83 (donor C). Figure 4 shows the contribution of phyla in the created stable systems, where the abundance of Actinobacteria, Proteobacteria and Verrucomicrobia did not significantly differ among the three donors based on a *p*-value of 0.05 among the three donors. Contrary to this, the abundance of Firmicutes and Bacteroidetes was different. Donor C did not show different abundances compared to donors A and B. When comparing donors A and B, A showed a decrease in Firmicutes and an increase in Bacteroidetes.

At the genus level, the genera *Bifidobacteria*, *Blautia*, *Faecalibacterium*, *Gemmiger*, *Lachnospiraceae*, *Parabacteroides*, *Roseburia* and *Ruminococcus* showed no significant differences among the three donors. Some differences were due to the lack of genera in the donor stool. *Dialister* was neither abundant in the stool nor in the stable system of donor B. Donor A lacked *Eisenbergiella*, *Oscillibacter* and *Prevotella*. In contrast, donor C showed a high abundance of *Prevotella*, while donor B only had a low abundance of this genera. For donor C, *Prevotella* was also abundant in a high number in the stable system. In the stable system of donor B, the abundance of *Akkermansia* was reduced, while *Clostridium XlVa* increased. The abundance of *Alistipes* was significantly different between donors A and B, with A showing higher values. For *Bacteroides* as well as for *Escherichia* and *Shigella*, donor C showed a reduced abundance compared to donor B.

In general, different donor stools led to different in vitro microbiomes regarding several factors. For cell count, propionate, and the phyla of Actinobacteria, Verrucomicrobia and Proteobacteria no differences were observed. The abundance of Firmicutes and Bacteroidetes was indeed divergent among the donors. Therefore, the metabolization of acetate, butyrate and isovalerate was different as well as diversity and richness. Nevertheless, the produced systems were comparable to in vitro growth, as shown by the formation of the stable system. As Figure 4 shows, the shift in the system towards a higher abundance of Bacteroidetes and a lower abundance of Firmicutes was common in all three donors. In general, the cultivation system has a huge impact on which system is formed, but the in vitro microbiome still has some characteristics originating from the donor stool.

### 3.4. Comparison of Different Donor Stools

When cultivating the microbiota in vitro, a high similarity with the original stool is the aim. For donor A, the cell count in the cultivated stable system differed: it increased significantly to 1 ± 0.6 × 10^8^ (facultative aerobe) and 0.9 ± 0.2 × 10^10^ CFU mL^−1^ (anaerobe). Regarding the metabolic profile, an increase was also observed. The amount of SCFAs in the broth was significantly higher with a value of 8.29 ± 0.55 mg mL^−1^ compared to the donor stool (6.80 ± 0.47 mg mL^−1^). These increases in cell count and metabolites are probably linked since more cells produce more metabolites. Regarding the composition of these cells, the abundance of Proteobacteria and Verrucomicrobia showed no significant difference between stool and cultivated broth. On the other hand, the phyla Actinobacteria, Bacteroidetes and Firmicutes differed significantly based on a *p*-value of 0.05 from the original sample. As can be seen in Figure 4, the abundance of Actinobacteria in stool was 2.02%, whereas it was lower in the cultivated system (0.09 ± 0.06%). Furthermore, the abundance of Firmicutes was decreased from the original value of 48.52% in the stool to an abundance of 17.13 ± 2.12% in the stable system. In contrast, the abundance of Bacteroidetes increased during cultivation from 46.89% (stool) to 76.10 ± 1.96% in the stable system. For donor B and C, the composition of the in vitro microbiota was similar and comparable to donor A. All three artificial systems showed significant increases in cell count and SCFAs with slight differences in the extent of increase. The microbial composition in the stable systems was defined by an increase in Bacteroidetes and Proteobacteria and a decrease in Firmicutes and Actinobacteria. Differences can only be observed for the phylum of Verrucomicrobia. For donors A and C, an increase in abundance was detected in the stable system. However, these observations represent only a trend but no significant differences. The microbial diversity in the cultivated system of donor A showed a significantly lower value (16.99 ± 1.01) than in the stool (21.42). However, the richness in the system was not affected. Here, the richness of 107 ± 3.94 in the stable state was comparable to that in the donor stool (100). The behavior of the cultivations of donors B and C was similar. Consequently, the established system is more influenced by the system parameters themselves. The behavior of the three donor stools during cultivation was comparable and similar. Nevertheless, individual donor characteristics remained.

In this study, differences among three donor stool samples were investigated. Three samples are considered as the scientific standard and commonly accepted in this field of research, as comparable studies used similar sample numbers. In other publications, the value ranges from four [36] and three [34] to even only two donor samples [24]. Nevertheless, it should be considered that the donor stools used in this study shared a common social and environmental background. We cannot exclude that this fact might lead to a similar in vitro microbiota system and prevented the detection of differences based on the donor stool. Additionally, the sample volume of three was sufficient to validate the concept, but more samples will be needed to confirm the findings presented and conclusions drawn here.

### 3.5. Comparison with the Inoculum

When comparing the in vitro microbiota with the fecal inoculum (Figure 4), all phyla for all donors showed significantly different abundances, except for the abundance of Verrucomicrobia. Additionally, diversity and richness showed similar values with no significant differences. Nevertheless, inocula similar to those prepared in this study, are used in current fecal microbiota transplantation concepts. Disregarding the high differences between stool and inoculum, the method showed a high recovery rate [62,63]. This indicates that in vitro microbiota, irrespective of the donor, could serve as a potential enhancement of FMT, since they show a higher similarity with the stool compared to the inoculum. However, it remains unknown how in vitro infusion material adapts to the individual human gut. Therefore, further experiments and testing will be required in order to close the gap of knowledge regarding the effectiveness of in vitro microbiota applied in FMT.

## 4. Conclusions

In the current study, a human in vitro intestinal microbiome of three different donors was developed, established and assessed. From the results obtained, we can conclude that the in vitro cultivation of a human intestinal microbiome is a valid alternative, but results in a slightly different system compared to in vivo. The number of anaerobic as well as facultative aerobic cells increased, as did the production of SCFAs. Regarding the microbial profile, the cultivation led to a decrease in the abundance of Actinobacteria. The abundance of Bacteroidetes increased, while Firmicutes decreased. Proteobacteria and Verrucomicrobia showed no significant changes. Diversity in the in vitro system decreased, but richness was not affected by cultivation. When cultivating stool from three different donors, the behavior of the created in vitro system was similar and comparable, but showed unique characteristics originating from the composition of the stool. The aim, however, was not to produce an exact copy of the in vivo system, but rather to demonstrate that a culturable, stable community with a high diversity and richness of representative gut bacteria can be produced. Further, the concentration and ratio of SCFAs were required to be comparable to the in vivo system to increase the chance of recovery when using the broth as an infusion material. As shown by this study, this can be reached, but the cultivation system and cultivation conditions have a major impact on the resulting microbiota profile. The produced in vitro microbiota can be influenced by choosing system parameters, which provide some degrees of freedom in producing a certain target microbiome, as well as the stool characteristics, with the cultivation parameters having a higher impact. Nevertheless, it should be considered that the donor stools used in this study shared a common social and environmental background. We cannot exclude that this fact might lead to a similar in vitro microbiota system and prevented the detection of differences based on the donor stool. Additionally, the sample volume of three was sufficient to validate the concept, but more samples will be needed to confirm the findings presented and conclusions drawn here. Consequently, further parameters, such as pH or stirring rate, as well as more and especially different stool samples from donors with different backgrounds, need to be cultivated and investigated to improve and enhance the system established here.

Comparing stool and the created system in regard to the fecal microbiota transplantation concept, we can conclude that the in vitro microbiome shows a higher similarity with the donor stool than the inoculum that is normally used for therapy. Nevertheless, future studies in vivo need to confirm the results, since it is not clear whether cultivated bacteria have the same impact on patients’ recovery as FMT itself.

## Figures and Tables

**Table 1:** Ingredients for preparation of 1000 mL Continuous Flow Fermentation Medium adapted according to Macfarlane and Gibson [43].

Stock Solution	Reagent	Manufacturer	Weight [g]	Dissolution in Double Distilled H_2_O [mL]
l	Casein peptone	Gerbu Biotechnik, Heidelberg, Germany	1.3	800
	Yeast extract	Gerbu Biotechnik, Heidelberg, Germany	2	
	NaHCO_3_	Bernd Kraft, Duisburg, Germany	2	
	CaCl_2_	Sigma-Aldrich, Saint-Louis, MO, USA	0.01	
	Pectin from citrus	Sigma-Aldrich, Saint-Louis, MO, USA	2	
	Xylan from beechwood	Iris Biotech, Marktredwitz, Germany	2	
	Arabinogalactan	Sigma-Aldrich, Saint-Louis, MO, USA	2	
	Starch	Merck KGaA, Darmstadt, Germany	5	
	Casein	Sigma-Aldrich, Saint-Louis, MO, USA	3	
	Inulin from Dahlia tubers	Sigma-Aldrich, Saint-Louis, MO, USA	1	
	NaCl	Carl Roth GmbH, Karlsruhe, Germany	0.75	
2	K_2_HPO_4_	Sigma-Aldrich, Saint-Louis, MO, USA	0.04	50
	KH_2_PO_4_	Sigma-Aldrich, Saint-Louis, MO, USA	0.04	
	MgSO_4_	Sigma-Aldrich, Saint-Louis, MO, USA	0.01	
3	Bile salts	Sigma-Aldrich, Saint-Louis, MO, USA	0.5	50
	L-Cysteine	Gerbu Biotechnik, Heidelberg, Germany	0.5	
4	Porcine gastric mucin (type II)	Sigma-Aldrich, Saint-Louis, MO, USA	4	100
5	Hemin solution	Sigma-Aldrich, Saint-Louis, MO, USA	10	-
6	Vitamin K1 solution	Alfa Aesar, Karlsruhe, Germany	0.2	-

**Table 2:** Characterization of the three donors by cell count, metabolites as well as richness, Shannon effective index and the ratio between Firmicutes and Bacteroidetes.

		Donor A	Donor B	Donor C
Cell count	Aerobic [10^5^ CFU mL^−1^]	0.7 ± 0.4	10 ± 9	9 ± 1
	Anaerobic [10^8^ CFU mL^−1^]	4 ± 3	2 ± 0.02	4 ± 0.6
Metabolic profile	Acetate [mg mL^−1^]	3.17 ± 0.21	2.74 ± 0.07	2.59 ± 0.54
	Propionate [mg mL^−1^]	2.00 ± 0.16	1.15 ± 0.04	1.02 ± 0.16
	Butyrate [mg mL^−1^]	1.48 ± 0.08	0.99 ± 0.03	2.14 ± 0.26
	Isovalerate [mg mL^−1^]	0.15 ± 0.02	0.24 ± 0.00	0.34 ± 0.04
	Σ SCFAs [mg mL^−1^]	6.80 ± 0.47	5.12 ± 0.14	6.09 ± 1.00
Microbial profile	Richness [-]	100	123	122
	Shannon effective index [-]	21.42	38.43	46.34
	Ratio F:B	1.03	1.13	2.60

**Table 3:** Evolution of microbial composition, richness and diversity of inoculum during cultivation of donor A.

		Processing Time				
	Inoculum	After Inoculation (<1.0 h)	8.3 ± 2.6 h	24.6 ± 0.6 h	48.0 ± 1.3 h	Stable System (>77.0 h)
Actinobacteria [%]	3.44 ± 0.83	3.16 ± 0.32	2.58 ± 1.01	2.27 ± 1.33	0.23 ± 0.02	0.09 ± 0.05
Bacteroidetes [%]	1.59 ± 1.09	0.90 ± 0.12	0.63 ± 0.67	37.91 ± 1.65	73.20 ± 1.46	75.99 ± 1.78
Firmicutes [%]	94.82 ± 0.75	95.78 ± 0.11	85.41 ± 7.62	49.20 ± 2.12	21.64 ± 1.69	16.92 ± 1.96
Proteobacteria [%]	0.12 ± 0.06	0.12 ± 0.14	11.37 ± 7.69	10.60 ± 3.81	3.83 ± 0.94	4.38 ± 2.42
Verrucomicrobia [%]	0.03 ± 0.01	0.04 ± 0.01	0.01 ± 0.01	0.02 ± 0.02	1.10 ± 0.76	2.22 ± 0.72
Richness [-]	103.40 ± 5.68	94.33 ± 10.02	11.00 ± 4.36	96.33 ± 12.66	95.00 ± 11.14	105.17 ± 5.71
Shannon effective index [-]	23.35 ± 2.76	18.69 ± 0.86	8.32 ± 4.23	24.21 ± 1.35	19.28 ± 1.65	17.28 ± 1.14

**Table 4:** Values of cell count, metabolic profile and microbial profile in the stable system (>77 h processing time) for the cultivation of the three donor stools.

		Donor A	Donor B	Donor C
Cell count	Aerobic [10^8^ CFU mL^−1^]	1 ± 0.6	2 ± 1	0.7 ± 0.07
	Anaerobic [10^10^ CFU mL^−1^]	0.9 ± 0.2	1 ± 0.3	1 ± 0.004
Metabolic profile	Acetate [ mg mL^−1^]	3.46 ± 0.22	3.82 ± 0.14	3.52 ± 0.05
	Propionate [ mg mL^−1^]	2.94 ± 0.13	2.94 ± 0.13	3.00 ± 0.07
	Butyrate [ mg mL^−1^]	1.64 ± 0.16	1.57 ± 0.16	2.39 ± 0.15
	Isovalerate [ mg mL^−1^]	0.24 ± 0.04	0.36 ± 0.08	0.31 ± 0.00
	Σ SCFAs [ mg mL^−1^]	8.29 ± 0.55	8.68 ± 0.51	9.22 ± 0.27
Microbial profile	Richness [-]	105.17 ± 5.71	96.00 ± 7.07	115.00 ± 2.83
	Shannon effective index [-]	17.28 ± 1.14	24.40 ± 1.75	21.56 ± 1.25

## Data Availability

Raw sequencing data are available at the Sequence Read Archive under accession number PRJNA719524.
